# Thresholds of socio-economic and environmental conditions necessary to escape from childhood malnutrition: a natural experiment in rural Gambia

**DOI:** 10.1186/s12916-018-1179-3

**Published:** 2018-11-01

**Authors:** Mayya Husseini, Momodou K Darboe, Sophie E Moore, Helen M Nabwera, Andrew M Prentice

**Affiliations:** 1 MRC Unit The Gambia at London School of Hygiene and Tropical Medicine, Atlantic Boulevard, Fajara, Banjul, The Gambia; 2 Regional Activity Centre for Sustainable Consumption and Production (SCP/RAC), Sant Pau Art Nouveau Site, Carrer Sant Antoni Maria Claret, 167, 08025 Barcelona, Spain; 3 Department of Women and Children’s Health, King’s College London, 10th Floor North Wing, St Thomas’ Hospital, Westminster Bridge Road, London, SE1 7EH UK; 4 Centre for Maternal and Newborn Health, Liverpool School of Tropical Medicine, Pembroke Place, Liverpool, L3 5QA UK

**Keywords:** Child growth, Stunting, Socioeconomic status, Gambia, WASH, Hygiene, Environmental, Enteropathy, Global health

## Abstract

**Background:**

Childhood malnutrition remains highly prevalent in low-income countries, and a 40% reduction in under-5 year stunting is WHO’s top Global Target 2025. Disappointingly, meta-analyses of intensive nutrition interventions reveal that they generally have low efficacy at improving growth. Unhygienic environments also contribute to growth failure, but large WASH Benefits and SHINE trials of improved water, sanitation and hygiene (WASH) recently reported no benefits to child growth.

**Methods:**

To explore the thresholds of socio-economic status (SES) and living standards associated with malnutrition, we exploited a natural experiment in which the location of our research centre within a remote rural village created a wide diversity of wealth, education and housing conditions within the same ecological setting and with free health services to all. A composite SES score was generated by grading occupation, education, income, water and sanitation, and housing and families were allocated to 5 groups (SES1 = highest). SES ranged from very poor subsistence-farming villagers to post graduate staff with overseas training. Nutritional status at 24 m was obtained from clinic records for 230 children and expressed relative to WHO Growth Standards.

**Results:**

Height-for-age (HAZ) and weight-for-age (WAZ) Z-scores were strongly predicted by SES group. HAZ varied from − 0.67 to − 2.23 (*P* < 0.001) and WAZ varied from − 0.90 to − 1.64 (*P* < 0.001), from SES1 to SES5, respectively. Weight-for-height (WHZ) showed no gradient. Children in SES1 showed greater dispersion so were further divided in a post hoc analysis. Children resident in Western housing on the research compound (SES1A) had HAZ = + 0.68 and WAZ = + 0.36. The residual gradient between those in SES1B and SES5 spanned only 0.65 Z-score for HAZ (− 1.58 to − 2.23) and was not significant for WAZ or WHZ.

**Conclusions:**

The large difference in growth between children in SES1A living in Western-type housing and SES1B children living in the village, and the very shallow gradient between SES1B and SES5, implies a very high SES threshold before stunting and underweight will be eliminated. This may help to explain the lack of efficacy of the recent WASH interventions and points to the need for what is termed ‘Transformative WASH’. Good quality housing, with piped water into the home, may be key to eliminating malnutrition.

## Background

Severe growth faltering leading to stunting and underweight remains highly prevalent in low- and middle-income countries [1] (LMICs) and is estimated to play a role in 45% of under-5 year child deaths [2]. An analysis of data from 54 such countries shows that babies are born small and then follow a remarkably uniform pattern of stunting initiated soon after birth and continuing to a nadir at about 24 months [1]. In Africa and SE Asia average height-for-age Z-scores (HAZ) fall to less than − 2 HAZ against the WHO Growth Reference Standard [1]. Weight-for-age Z-scores (WAZ) vary more widely between populations.

Despite many decades of research, the precise aetiology of this growth failure remains obscure. It is ecologically associated with a number of correlates of poverty including generally poor diets of low diversity; food insecurity; poor water supply, sanitation and hygiene; frequent infections and inflammation (including enteric infections that cause a persistent environmental enteropathy that may inhibit nutrient uptake); and constraints on mothers’ time combined with poor parental understanding of the principles of childcare. However, amelioration of some of these factors frequently has a disappointing impact on child growth. For instance, during 35 years of intensive clinical and nutritional interventions in rural Gambia, we have reduced child mortality rates over 10-fold [3] and observed a profound decline in diarrhoeal disease [4], yet the prevalence of stunting is still 30% [5]. Others have shown that careful evaluation of nutritional and educational interventions either singly or in combination achieve improvements in HAZ of less than 0.3 Z-scores [6]; representing less than one sixth of the average deficit.

The excellent prenatal and post-natal growth shown by the cohorts of carefully selected children used to derive the INTERGROWTH Fetal Growth [7], and WHO Child Growth Standards [8] demonstrate the potential for excellent growth when the environmental conditions are right. Furthermore, rates of stunting decline quite rapidly as countries pass through the demographic transition. [9]. So why is it so difficult to improve child growth in low-income settings? The current paradigm is that multiple interventions must be introduced simultaneously [10] and numerous trials combining efforts to improve water, sanitation, hygiene (WASH), diet, breast-feeding rates, infant stimulation and parental education are in progress. Notably, the large-scale WASH Benefits trials in Kenya and Bangladesh have recently reported their outcomes with respect to stunting [11, 12]. The intervention arms that promoted infant and young child feeding (IYCF) showed a significant reduction in stunting but with a very modest effect size (0.13–0.16 Z-score for length in Kenya and 0.13–0.25 Z-score in Bangladesh). The SHINE Trial in Zimbabwe has also reported significant but limited success with IYCF (0.16 Z-score for length) [13]. Disappointingly, none of the WASH interventions reduced stunting.

In this study, we exploited a natural experiment to examine thresholds of socio-economic development associated with growth faltering. Our research centre at MRC Keneba is embedded within a rural subsistence-farming Gambian village. The centre employs over 200 indigenous staff ranging from scientists, physicians and senior management, through nurses, laboratory technicians and fieldworkers to lower grades of support staff such as cleaners many of whom are natives of Keneba or nearby villages. We also studied villagers without any paid employment. These circumstances have created a very wide socio-economic and educational gradient within the same confined ecological setting and allowed us to examine the effects of these gradients on child growth.

## Methods

### Study location

The UK Medical Research Council has maintained a research presence in the poor rural West Kiang region of The Gambia, since 1948. The field station comprises a self-sufficient 4-acre site with housing, laboratories, clinic and administrative buildings contained within a perimeter fence. All housing is of ‘Western’ standard with tiled floors, metal roofs and suspended ceilings, fully plumbed kitchens and bathrooms with flushing toilets and hot and cold water to basins and showers, screened and louvered windows, gas cooking, fans and air conditioning. Water and electricity are available constantly and are not charged to staff, thus allowing unlimited usage. The keeping of livestock is strongly discouraged in the compound. Originally sited on the edge of the village, it has now become engulfed by new village compounds as the population has expanded. Most villagers rely for their livelihoods on subsistence farming, occasionally supplemented by some petty trading, and increasingly by remittances from family members who have migrated to urban areas or abroad. The village has a primary and middle school and an Islamic school. Uptake of education has increased in the past two decades, initially male dominated but now more equitable due to universal free education for girls. The MRC compound and the village are both supplied by clean tube-well water. Villagers obtain their water from a series of 30+ standpipes distributed as equitably as possible around the village. Water is potable as it emerges from the taps. Subsequent contamination during collection and storage is possible.

### Healthcare

For over 35 years, we have provided an exceptional level of free healthcare for villagers and staff (summarised in Box 1). Thus, availability of healthcare is constant across all socio-economic and educational levels, and for staff and villagers alike, and uptake is only determined by parental health-seeking behaviours. All women receive regularly scheduled ante-natal care, and their infants have a post-natal check-up and are called to well-baby clinics at 6 week, 3 m and then at 3 m intervals until 2 years of age. All children receive their full schedule of Extended Programme of Immunisation (EPI) vaccines and vitamin A supplementation according to WHO guidelines.

### Eligibility and sampling

The greatest variance in SES was associated with MRC employment. Using the Kiang West Demographic Surveillance System (DSS) [14], a list of all children with anthropometric data (length and weight measurements) taken at 24 ± 4 m of age and born between January 1993 and December 2009 was created. The sample size was determined by the number of MRC employees who had children at 24 m within the study window. All MRC employees on the list were approached to participate. This yielded 51 families and 133 children. This list was supplemented with data from 129 village children selected at random from our DSS database and matched by year of measurement. Children with any condition known to affect growth were excluded.

### Assessment of parental SES

A total of 98 parents were interviewed during the data collection period. Parental SES was evaluated using a modified version of a 43-item questionnaire originally developed to study the relationship between parental SES and child mortality elsewhere in The Gambia [15]. The questions were grouped according to occupation, income and possessions, education, access to water and sanitation, and housing. In the absence of indications to the contrary, each of these factors was assumed to have an equal impact on child growth; thus, each category was given the same weight of a maximum of 10 points. Each question within each category was then assigned an appropriately weighted score based on information from senior staff at the MRC about the perceived socio-economic value of items in the questionnaire. The aggregate SES score for each set of parents was then determined by adding up the 5 separate scores obtained for each category, giving a total maximum of 50 points. Initially the total parental scores were assigned a priori to 5 SES groups as follows: SES1 = > 25; SES2 = 21–25; SES3 = 17–21; SES4 = 14–17; and SES5 = < 14. It was subsequently noted that SES1 showed a wide dispersion of anthropometric values so this group was further divided as follows: SES1A = > 25 points and resident in Western-style housing within the MRC compound; and SES1B = > 25 points and resident in the village. The headline descriptors of each SES category are listed in Table 1.

**Table 1:** Typical SES characteristics of the five pre hoc determined groups and subsequent post hoc division of group 1

Pre hoc assignment	1A	1B	2	3	4	5
*N*	8	11	46	72	65	28
Occupation	One or both parents with senior MRC jobs; (e.g. scientific officer and admin staff)		One parent with middle grade job at MRC (e.g. field worker); Mother likely to be housewife	One farmer parent, other with lower grade job at MRC (e.g. cleaner)	Farmer parents	Farmer parents; father likely to be retired
Education	Both parents have > 10 years at English school. Most families have one parent with university qualifications and/or post graduate diplomas from abroad		Fathers have > 10 years at Arabic and English school; Mother likely to have approx. 5 years at English school	One parent with approx. 5 years in English school	Approx. 10 years for father and 5 years for mother in Arabic school	Approx. 5 years in Arabic school for father only
Income	Main income from MRC employment		Main income from MRC employment	Main income from MRC employment, remittances and minor produce sales	Dependent on minor produce sales and remittances	Dependent on remittances
Water and sanitation	Plumbed hot and cold water to kitchens and bathrooms, indoor flushing toilets, modern cooking facilities	Public tap for water, pit latrine exclusive to household, cook with firewood	Public tap for water, pit latrine exclusive to household, cook with firewood	Public tap for water, pit latrine exclusive to household, cook with firewood	Public tap for water, pit latrine exclusive to household, cook with firewood	Public tap for water, pit latrine shared with other households, cook with firewood
Housing	European grade cement housing with ceramic tile flooring, screened windows, fans and air conditioning, located on MRC compound	Local housing, cement as main material. Corrugated iron roofing. Occasional solar electricity. Unscreened windows, located in the village	Local housing, cement as main material. Corrugated iron roofing. Occasional solar electricity. Unscreened windows, located in the village	Local housing, cement as main material. Corrugated iron roofing. Occasional solar electricity. Unscreened windows, located in the village	Local housing, cement as main material. Corrugated iron roofing. Occasional solar electricity. Unscreened windows, located in the village	Basic housing; mud as main material. Corrugated iron or thatched roofing

### Anthropometry

In this population, anthropometric status declines rapidly in the first and second years of life and then remains somewhat stable before recovering slightly in later childhood [16]. We therefore obtained clinic records for heights and weights at 24 ± 4 m. Weight (in minimal clothing) and recumbent length (height) were recorded by trained anthropometrists using standard techniques and regularly calibrated apparatus. Appropriate weight data was found for 262 children, but there were 32 missing heights. The data were then restricted to those that had full anthropometric values available; yielding a final sample size of 230. HAZ, WAZ and WHZ were calculated using WHO Anthro software (version 3.2.2) based on the 2006 WHO Child Growth Standards [8]. Within the narrow age range selected for this study, there was no influence of age on any of the anthropometric measures. There was an influence of sex, so this has been included in the analysis model. Stunting was defined as HAZ < − 2, underweight as WAZ < − 2 and wasting as WHZ < − 2. Parental heights were obtained from our DHSS database. There were 2 missing maternal heights.

### Data analysis

Multi-level linear analysis was performed on anthropometric attainment at 24 m according to the 5 (later 6) SES groups with adjustment for parental heights and with intra-household clustering accounted for by including mother’s ID on the basis that children of this age live with their mothers. Date of measurement was included in the model to test for any secular drift but was not significant so was not included in the final analysis. All analyses were performed using the ‘Linear Models’ function in DataDesk version 7.0.2 (Data description Inc., Ithaca, NY).

## Results

On average fathers had 2 wives (range 1–4) and 12 children (range 1–33). Mothers averaged 6 children (range 1–12) and household size averaged 13 (range 4–27). Fathers averaged 3.5 years of formal education (range 0–18 years) and 8.0 years of Arabic/Islamic studies (range 0–24). Mothers averaged 2.0 years of formal education (range 0–16 years) and 4.4 years of Arabic/Islamic studies (range 0–13). Reported incomes ranged from less than US$50 to over US$20,000 per annum. Eight percent of families owned a car, 12% a motorcycle and 55% a bicycle. Seventeen percent had a refrigerator, 32% had a television and 19% had electricity at home. Housing ranged from very basic mud block huts to Western-style housing with hot and cold running water, showers, flushing toilets, gas cooking, refrigerators, mosquito screens, fans and air conditioning.

The mean anthropometric statistics for the whole sample were HAZ (% stunted), boys = − 1.94 (40%), girls = − 1.55 (35%) (*p* = 0.018 for sex); WAZ (% underweight) boys = − 1.46 (31%), girls = − 1.32 (21%) (*p* = 0.052 for sex); and WHZ (% wasted) boys = − 0.64 (5%), girls = − 0.68 (6%) (NS for sex). Notably, The Gambian children’s heights and weights at 2 years of age were very similar to the average heights and weights of WHO reference children at 1 year of age.

**Fig. 1:**
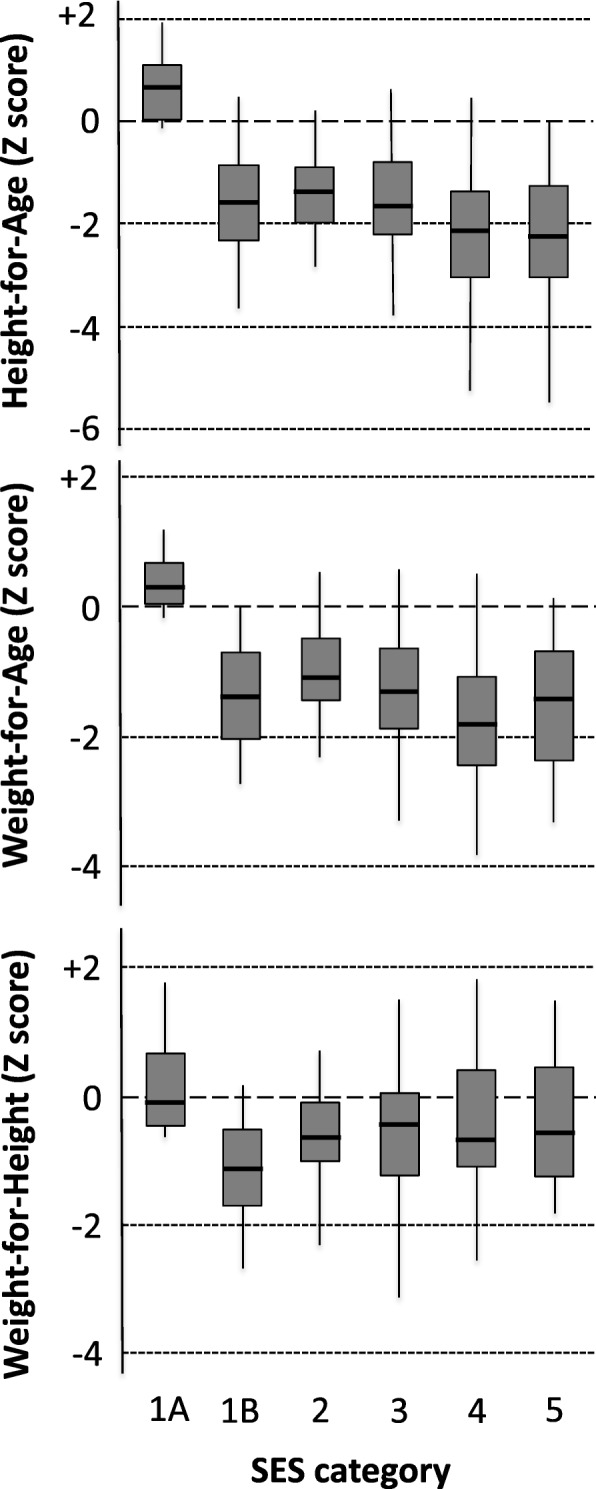
Means, interquartile ranges and 95% confidence intervals for anthropometric scores at 24 m. Sample sizes, tests for trends and individual group comparisons by Scheffé’s post hoc test are listed in Tables 2 and 3

Table 2 details the mean anthropometric Z-scores according to the 5 pre-determined SES groupings (see Fig. 1 for the interquartile ranges). HAZ varied strongly with SES with a range from − 0.67 in SES1 to − 2.23 in SES5 (*P* < 0.001); the proportions of stunted children were 26 and 54% respectively. Surprisingly, the trend between SES2, defined as upper-middle SES, and the lowest category of SES5 only spanned 0.65 Z-scores; and the proportion stunted varied from 37 to 54%. WAZ varied somewhat less strongly with SES with a range from − 0.90 in SES1 to − 1.64 in SES5 (*P* < 0.001); the proportions of underweight children were 31 and 43% respectively. The range in WAZ between SES2 and SES5 was only 0.44, and the proportions of underweight children were 17 and 43%. WHZ did not vary at all across SES groupings.

**Table 2:** Mean anthropometric scores at 24 m according to the 5 pre hoc SES categories

Variable	Mean per SES category					*P* value for trend
	1 upper	2 upper-middle	3 middle	4 lower-middle	5 lower	
*N*	19	46	72	65	28	
HAZ score	− 0.67 ^3,4,5^	− 1.58	− 1.69 ^1^	− 2.16 ^1^	− 2.23 ^1^	< 0.001
% stunted	26	37	44	54	54	
WAZ score	− 0.90	− 1.20	− 1.35	− 1.67	− 1.64	< 0.001
% underweight	31	17	19	34	43	
WHZ score	− 0.63	− 0.56	− 0.63	− 0.77	− 0.68	0.255
% wasted	10	4	4	9	4	
Mothers’ heights	159.6	162.0	164.0	161.3	161.9	0.014
Fathers’ heights	173.5 ^4^	171.3 ^4^	170.6	167.8 ^1,2^	170.4	< 0.001

Superscripts indicate the SES groups against which values are significantly different at *P* < 0.01 by Scheffé’s post hoc test. For the children’s anthropometry, their parents’ heights were included in the model. Child sex was also included in the model

Inspection of the data revealed a greater dispersion of HAZ and WAZ in SES1 (composed entirely of the more senior MRC staff) than the other groups suggesting that it remained a heterogeneous grouping that would merit further sub-division. We therefore performed a post hoc split of SES1 using the factor that seemed most likely to offer discriminatory power; namely whether or not the staff were resident within the MRC’s fenced compound (and therefore living in better quality accommodation) (SES1A) or in the village (SES1B) (see Table 1 for descriptors). The analysis by the 6 groupings is listed in Table 3 and illustrated with interquartile ranges in Fig. 1. The children brought up in the MRC compound had HAZ = + 0.68, WAZ = + 0.36 and WHZ = − 0.03. None were stunted or underweight, and 1 was very marginally wasted. In SES1B, where the families lived in the village, mean HAZ was − 1.65, mean WAZ was − 1.64 and mean WHZ was − 1.07 despite their very high SES. Children in SES1A were significantly taller and heavier than children from each of the other groups even when parental heights were included in the model (Scheffé’s post hoc tests, see Tables 2 and 3). WHZ did not differ between any of the groups.

**Table 3:** Mean anthropometric scores at 24 m according to 6 post hoc SES categories

Variable	Mean per SES category						*P* value for Trend
	1A upper	1B upper	2 upper-middle	3 middle	4 lower-middle	5 lower	
*N*	8	11	46	72	65	28	
HAZ score	0.68 ^1B,2,3,4,5^	− 1.65 ^1A^	− 1.58 ^1A^	− 1.69 ^1A^	− 2.16 ^1A^	− 2.23 ^1A^	< 0.001
% stunted	0	45	37	44	54	54	
WAZ score	0.36 ^1B,2,3,4,5^	− 1.64 ^1A^	− 1.20 ^1A^	− 1.35 ^1A^	− 1.67 ^1A^	− 1.64 ^1A^	< 0.001
% underweight	0	55	17	19	34	43	
WHZ score	− 0.03	− 1.07	− 0.56	− 0.63	− 0.77	− 0.68	0.255
% wasted	12	9	4	4	9	4	
Mothers’ heights	161.2	158.5	162.0	164.0	161.3	161.9	0.019
Fathers’ heights	172.6	174.1 ^4^	171.3	170.6	167.8 ^1B^	170.4	< 0.001

Superscripts indicate the SES groups against which values are significantly different at *P* < 0.01 by Scheffé’s post hoc test. For the children’s anthropometry, their parents’ heights were included in the model. Child sex was also included in the model

Parental heights were correlated with offspring HAZ (mother’s coefficient 0.66 (SE 0.13) Z-score per 10 cm (*P* < 0.0001); father’s coefficient 0.52 (SE 0.15) Z-score per 10 cm (*P* < 0.0001). There was no difference in maternal heights across the 5 or 6 SES groups. Paternal heights in SES1 and 2 were greater than in SES4. When SES1 was subdivided, it was the father’s in SES1B who were tallest and significantly taller than SES4. Father’s heights in SES1A were not significantly different to any other group.

## Discussion

The major WASH Benefits trials in Kenya and Bangladesh [11, 12] and the SHINE trial in Zimbabwe [13] have recently reported that promotion of improved water, supply, sanitation and handwashing had no effect on linear growth. These very disappointing results from large-scale, rigorously implemented trials in appropriate target populations require an explanation. Our current study suggests that the intent was correct but that the environmental hygiene threshold that permits normal child growth is far higher than achieved in WASH Benefits and SHINE. The original selection strategy of participants for the WHO MGRS [17] that forms the basis of the current WHO Growth Standards implicitly recognised this same point.

The key feature of this study is that the participant families had identical access to health care and, by virtue of living in the same small village, shared common exposures to many other health-determining factors. This allowed us to interrogate the influence of 5 socio-economic domains (occupation, income and possessions, education, access to water and sanitation, and housing) with far fewer confounders than would normally exist in such analyses. We were also able to examine a very steep gradient ranging from the poorest village families with very few resources to families in which one or both parents had lived and trained in high-income countries and had advanced educational qualifications and incomes that were several orders of magnitude higher than the villagers. Growth data was routinely measured and recorded for all children in the same anthropometric clinic, enabling an unbiased analysis of their attained size at 2 years of age; the growth nadir in this population.

The highly significant gradient in HAZ and WAZ across SES groups is unsurprising and validates the relevance of the SES metrics. The remarkable feature of the data is that the only children growing close to the WHO Growth Standard were those living in Western-type accommodation within the MRC campus (HAZ + 0.68, WAZ + 0.36, WHZ − 0.03). The children of staff of almost similar rank who, by virtue of a shortage of housing on the campus, lived in rented housing in the village fared substantially less well (HAZ − 1.65, WAZ − 1.64, WHZ − 1.07). The HAZ and WAZ advantage of children in SES1A was not explained by differences in parental heights suggesting that it is unlikely to reflect an inter-generational advantage.

Our analysis is unique to date and has the great strength of removing the influence of the healthcare and environmental variables listed in Box 1 that are concordant across all families and that otherwise would confound interpretation of the effects of the SES gradient studied. Its chief weakness is the limited sample size, and replication is needed if a similar natural experiment can be found elsewhere. The sample size limitation does not limit the key observation that child growth was far from desirable even in families with high levels of education, very good incomes and absolute food security. In a comparison of group SES1A and SES2 combined (*n* = 57) against the SES4 and SES5 groups combined (*n* = 93) HAZ varied by only 0.59 Z-scores from − 1.59 to − 2.18 and the prevalence of stunting from 39 to 54%. WAZ varied by only 0.38 Z-scores from − 1.28 to − 1.66. WHZ did not differ. An additional weakness is that the SES data were collected up to 15 year after the anthropometric data. This is of limited concern because we allocated occupation and salary ranking in MRC staff according to their grade when their child was 24 m and social mobility is very slow in villagers. Additionally, any misclassification would tend to nullify associations rather than introduce bias.

The modifiable components of children’s growth (i.e. the non-genetic components) are influenced by a myriad of nutrition-specific factors (breast-feeding and complementary feeding practices, food availability and quality, and parental knowledge of the principles of child feeding) and nutrition-sensitive factors (sanitation and hygiene, access to health care, infections and stress). This likely explains why interventions aimed solely at improving complementary feeding, and diet have shown limited efficacy, even when conducted under the rigorously controlled conditions of research trials. Meta-analysis of trials involving feeding and/or educational interventions demonstrate average HAZ responses of less than 0.3 Z-scores; equivalent to about one sixth of the usual deficit by 2 years of age in LMICs [6]. Following these disappointments, it was argued that the quality of nutrition interventions had to be improved still further with the provision of a comprehensive range of micro- and macro-nutrients, and large trials of lipid-based nutritional supplements have recently been completed. Unfortunately, these have, by and large, also yielded disappointing outcomes ranging from no significant impact [18–21] to maximum benefits again not exceeding 0.3 Z-scores for height [22, 23]. Very recently, the WASH Benefits and SHINE trials have again confirmed this result. The groups receiving promotion of the WHO IYCF guidelines showed improvements of 0.13 (Kenya) and 0.25 (Bangladesh) Z-score in the nutrition group and of 0.16 (Kenya) and 0.13 (Bangladesh) Z-score in the nutrition plus WASH groups against the average HAZ in the control groups of − 1.54 in Kenya and − 1.79 in Bangladesh [11, 12]. The SHINE trial has recently reported very similar results with an improvement of 0.16 for HAZ in the IYCF group and 0.02 in the IYCF+WASH against a background level of − 1.59 Z-scores in the standard of care group [13].

A chief target of the WASH interventions has been to ameliorate the gut damage that is caused by poor living conditions [10]. Our previous work in The Gambia identified persistent and chronic damage to the gut (now commonly termed environmental enteric disease (EED)) as a likely mediating factor in growth failure [24, 25]. Enteropathy impairs nutrient absorption, increases energy and nutrient losses and causes chronic inflammation that suppresses the growth hormone/IGF1 somatotrophic axis [10, 26]. This chronic gut damage affects most children in poor settings and is believed to be initiated and sustained by repeated exposure to microorganisms (and possibly other aggravating factors) in a contaminated environment. A study in Bangladesh has found differences in biomarkers of EED between ‘clean’ and ‘dirty’ households and a 0.54 HAZ difference in growth in favour of the clean households, though it should be noted that average growth was still very poor at − 1.66 HAZ [27].

Overcoming such influences has formed the rationale for numerous WASH interventions. However, a Cochrane systematic review and meta-analysis of such trials published in 2013 concluded that efficacy was poor [28]. The analysis of randomised trials including over 4600 children studied over 9–12 m intervention periods revealed no evidence of any beneficial effect on weight-for-age or weight-for-height and only a marginally significant impact on height-for-age of less than one tenth of a standard deviation (+ 0.08 Z-score, 95%CI 0.00–0.16). An individual participant data analysis (*n* = 5375) was conducted for 5 RCTs. For HAZ, this identified a significant age interaction with children under 2 years gaining most benefit. Nonetheless, the benefit in this selected age band was still only 0.25 (95% CI 0.14–0.36) Z-scores [28]. The WASH Benefits and SHINE results are even more discouraging insofar as their null outcomes arose from very large, rigorously implemented trials in appropriate target populations [10–12].

One hypothesis about the lack of effects of the WASH interventions in WASH Benefits and SHINE is that they did not target the whole community; they only worked within individual households. Therefore, spillovers from the un-intervened households could still contribute to infection and inflammation in the study children. There is evidence that community-wide total sanitation interventions may have bigger effects [29–31], possibly related to herd protection. A similar effect may be at play within the MRC compound though it should be stressed that there is free movement into and out of the compound with multiple opportunities for cross-exposure between the SES1A children and villagers through social visits, markets, clinics and by sharing of transport.

The emergent consensus is that it has proven extremely difficult to shift child growth patterns by more than one sixth of the total deficit that accrues by age 2 years in LMICs. The Lancet Series on Maternal and Child Nutrition reinforces the conclusion that nutrition-related interventions will have limited impact [32]. The authors estimated that, even if scaled up to 90% coverage, the implementation of all of the currently identified evidence-based nutrition and nutrition-related interventions would eliminate around 20% of stunting globally.

The current study suggests a resolution to this paradox. Our data indicates that there is a very high threshold of living conditions that has to be surpassed for stunting to resolve. Our evidence suggests that despite adequate parental knowledge of the principles of hygiene and good nutrition within SES1B and SES2, and despite income levels allowing access to moderately good housing and virtually any foods, the children of such families still show profound growth faltering in early life. An explanation might lie in two domains. First, there are many fewer animals within the MRC compound and they are kept out of the houses, so it is much easier for parents to maintain their children in a setting with minimal faecal contamination; a factor considered a likely driver of infections and enteropathy [10, 33]. Second, studies have found that the volume of water usage is highly dependent upon time taken to access the water [33]. Even if water is available at an adjacent standpipe requiring just 2 min for collection, water usage has been found to average 20 l per person per day, whereas usage rises to 60 l per person per day when water is piped directly into the home [34]. We propose that the disappointing outcomes from the WASH trials [10–13] have arisen, not because the theoretical premise behind the trials is incorrect, but because the interventions have fallen short of the very high threshold indicated by our current data.

## Conclusions

On the basis of these observations, we infer that improvements in housing conditions including, and perhaps especially, the provision of piped water into the home, may be a key factor in explaining why national stunting rates remain so persistent in low-income settings yet decline so markedly as countries pass through the economic transition. This could be tested using cluster-randomised or step-wedge intervention designs of what we term WASH++ (and others are terming ‘Transformative WASH’) and, if true, would lead to a major shift in priorities for implementation of nutrition-specific and WASH-type interventions.

## **Box 1**: Concordant factors across the population sample

- Residence within the same 4 km^2^
- Free access to MRC Keneba Clinic providing excellent healthcare (3 qualified medical doctors, diagnostics labs, etc.) 5 days per week. Round-the-clock (24/7) emergency access to on-call staff.
- Free access to medications with reliable supply. Close to 100% vaccination and vitamin A supplementation rates using full WHO schedule. Guaranteed cold chain. Ready access to oral rehydration fluids.
- Equal access to 3-monthly growth monitoring. All children called to 3-monthly healthy child clinics.
- Free referral to secondary and tertiary health care when required. Free transportation provided. Free nutritional rehabilitation according to WHO protocols for any child falling below − 3 WHZ.
- Universal access to excellent ante-natal and delivery care including provision of blanket FeFol supplementation and malaria prophylaxis in pregnancy.
- Universal breast-feeding to at least 18 m for village children and similar for staff children. No formula milk available in local markets. Keneba is a Baby Friendly Community supported by the National Nutrition Agency with promotion of exclusive breast-feeding to 6 m.
- Water supplied from the same borehole and via same storage tank.

## Abbreviations

HAZ: Height-for-age Z-score
LSHTM: London School of Hygiene & Tropical Medicine
MRC: Medical Research Council
SES: Socio-economic status
WASH: Water, sanitation and hygiene
WAZ: Weight-for-age Z-score
WHO: World Health Organisation
WHZ: Weight-for-height Z-score

## References

[1] Victora CG, de Onis M, Hallal PC, Blössner M, Shrimpton R (2010). Worldwide timing of growth faltering: revisiting implications for interventions. Pediatrics.

[2] Black RE, Victora CG, Walker SP (2013). Maternal and child undernutrition and overweight in low-income and middle-income countries. Lancet.

[3] Rayco-Solon P, Moore SE, Fulford AJ, Prentice AM (2004). Fifty-year mortality trends in three rural African villages. Tropical Med Int Health.

[4] Poskitt EM, Cole TJ, Whitehead RG (1999). Less diarrhoea but no change in growth: 15 years’ data from three Gambian villages. Arch Dis Child.

[5] Nabwera HM, Fulford AJ, Moore SE, Prentice AM (2017). Growth faltering persists in rural Gambian children despite four decades of intervention. Lancet Global Health.

[6] Dewey KG, Adu-Afarwuah S (2008). Systematic review of the efficacy and effectiveness of complementary feeding interventions in developing countries. Matern Child Nutr..

[7] Papageorghiou AT, Ohuma EO, Altman DG (2014). International standards for fetal growth based on serial ultrasound measurements: the Fetal Growth Longitudinal Study of the INTERGROWTH-21st project. Lancet.

[8] World Health Organisation (2006). WHO child growth standards based on length/height, weight and age. Acta Paediatr Suppl.

[9] Stevens GA, Finucane MM, Paciorek CJ, Flaxman SR, White RA, Donner AJ, Ezzati M, and on behalf of Nutrition Impact Model Study Group (Child Growth) (2012). Trends in mild, moderate, and severe stunting and underweight, and progress towards MDG 1 in 141 developing countries: a systematic analysis of population representative data. Lancet.

[10] Humphrey JH, Jones AD, Manges A, Mangwadu G, Maluccio JA, Mbuya MN, Moulton LH, Ntozini R, Prendergast AJ, Stoltzfus RJ, Tielsch JM, Sanitation Hygiene Infant Nutrition Efficacy (SHINE) Trial Team (2015). The sanitation hygiene infant nutrition efficacy (SHINE) trial: rationale, design, and methods. Clin Infect Dis.

[11] Null C, Stewart CP, Pickering AJ (2018). Effects or water quality, sanitation and handwashing, and nutritional interventions on diarrhoea and child growth in rural Kenya: a cluster-randomised trial. Lancet Global Helath.

[12] Luby SP, Rahman M, Arnold BF (2018). Effects or water quality, sanitation and handwashing, and nutritional interventions on diarrhoea and child growth in rural Bangladesh: a cluster-randomised trial. Lancet Global Helath.

[13] Humphrey JH, Mbuya MNN, Ntozini R, et al. Prendergast AJ for The Sanitation Hygiene Infant Nutrition Efficacy (SHINE) Trial Team. Independent and combined effects of improved water, sanitation and hygiene, and improved complementary feeding, on child stunting and anaemia in rural Zimbabwe: a cluster-randomised trial. Lancet Global Health. 2018. In press. 10.1016/S2214-109X(18)30374-7 PMC6293965 30554749

[14] Hennig BJ, Unger SA, Dondeh BL, Hassan J, Hawkesworth S, Jarjou L, Jones KS, Moore SE, Nabwera HM, Ngum M, Prentice A, Sonko B, Prentice AM, Fulford AJ. Cohort profile: the Kiang West Longitudinal Population Study (KWLPS)—a platform for integrated research and health care provision in rural Gambia. Int J Epidemiol. 2015. 10.1093/ije/dyv206. 10.1093/ije/dyv206 PMC5837564 26559544

[15] Ratcliffe AA, de Savingy D (2005). Parent’s socio-economic status and social support as risks for child mortality: consideration of health equity in The Gambia. Measuring Health Equity in Small Areas: Findings from Demographic Surveillance Systems INDEPTH network.

[16] Prentice AM, Ward KA, Goldberg GR, Jarjou LM, Moore SE, Fulford AJ, Prentice A (2013). Critical windows for nutritional interventions against stunting. Am J Clin Nutr.

[17] de Onis M, Garza C, Victora CG, MK BB, Norum KR (2004). The WHO multicentre growth reference study (MGRS): rationale, planning, and implementation. Food Nutr Bull.

[18] Thakwalakwa CM, Ashorn P, Jawati M, Phuka JC, Cheung YB, Maleta KM (2012). An effectiveness trial showed lipid-based nutrient supplementation but not corn-soya blend offered a modest benefit in weight gain among 6- to 18-month-old underweight children in rural Malawi. Public Health Nutr.

[19] Maleta KM, Phuka J, Alho L (2015). Provision of 10-40 g/d lipid-based nutrient supplements from 6 to 18 months of age does not prevent linear growth faltering in Malawi. J Nutr.

[20] Ashorn P, Alho L, Ashorn U (2015). Supplementation of maternal diets during pregnancy and for 6 months postpartum and infant diets thereafter with small-quantity lipid-based nutrient supplements does not promote child growth by 18 months of age in rural Malawi: a randomized controlled trial. J Nutr.

[21] Mangani C, Maleta K, Phuka J, Cheung YB, Thakwalakwa C, Dewey K, Manary M, Puumalainen T, Ashorn P (2015). Effect of complementary feeding with lipid-based nutrient supplements and corn-soy blend on the incidence of stunting and linear growth among 6- to 18-month-old infants and children in rural Malawi. Matern Child Nutr.

[22] Iannotti LL, Dulience SJ, Green J, Joseph S, François J, Anténor ML, Lesorogol C, Mounce J, Nickerson NM (2014). Linear growth increased in young children in an urban slum of Haiti: a randomized controlled trial of a lipid-based nutrient supplement. Am J Clin Nutr.

[23] Hess SY, Abbeddou S, Jimenez EY (2015). Small-quantity lipid-based nutrient supplements, regardless of their zinc content, increase growth and reduce the prevalence of stunting and wasting in young Burkinabe children: a cluster-randomized trial. PLoS One.

[24] Lunn PG, Northrop-Clewes CA, Downes RM (1991). Intestinal permeability, mucosal injury, and growth faltering in Gambian infants. Lancet.

[25] Campbell DI, Elia M, Lunn PG (2003). Growth faltering in rural Gambian infants is associated with impaired small intestinal barrier function, leading to endotoxemia and systemic inflammation. J Nutr.

[26] Humphrey JH (2009). Child undernutrition, tropical enteropathy, toilets, and handwashing. Lancet.

[27] Lin A, Arnold BF, Afreen S, Goto R, Huda TM, Haque R, Raqib R, Unicomb L, Ahmed T, Colford JM, Luby SP (2013). Household environmental conditions are associated with enteropathy and impaired growth in rural Bangladesh. Am J Trop Med Hyg.

[28] Dangour AD, Watson L, Cumming O, Boisson S, Che Y, Velleman Y, Cavill S, Allen E, Uauy R (2013). Interventions to improve water quality and supply, sanitation and hygiene practices, and their effects on the nutritional status of children. Cochrane Database Syst Rev.

[29] Fuller JA, Villamor E, Cevallos W, Trostle J, Eisenberg JN (2016). I get height with a little help from my friends: herd protection from sanitation on child growth in rural Ecuador. Int J Epidemiol.

[30] Harris M, Alzua ML, Osbert N, Pickering A (2017). Community-level sanitation coverage more strongly associated with child growth and household drinking water quality than access to a private toilet in rural Mali. Environ Sci Technol.

[31] Pickering AJ, Djebbari H, Lopez C, Coulibaly M, Alzua ML (2015). Effect of a community-led sanitation intervention on child diarrhoea and child growth in rural Mali: a cluster-randomised controlled trial. Lancet Glob Health.

[32] Bhutta ZA, Das JK, Rizvi A, Gaffey MF, Walker N, Horton S, Webb P, Lartey A, Black RE, Lancet Nutrition Interventions Review Group; Maternal and Child Nutrition Study Group (2013). Evidence-based interventions for improvement of maternal and child nutrition: what can be done and at what cost?. Lancet.

[33] Korpe PS, Petri WA (2012). Environmental enteropathy: critical implications of a poorly understood condition. Trends Mol Med.

[34] Cairncross S, Feachem R (1993). Environmental health engineering in the tropics: an introductory text.

